# Examining the relationship between physical literacy and resilience against COVID-19-induced negative mental states in Chinese adolescents

**DOI:** 10.1186/s12889-024-18842-x

**Published:** 2024-06-29

**Authors:** Jiarun Wu, Garry Kuan, Yishuai Wang, Zhutang Liu, Xiaoyu Hu, Yee Cheng Kueh, Xinding Zhang

**Affiliations:** 1 School of Physical Health, Guizhou University of Traditional Chinese Medicine, Guiyang, Guizhou China; 2 Exercise and Sports Science Programme, School of Health Sciences, Universiti Sains Malaysia, 16150 Kubang Kerian, Kelantan, Malaysia; 3 Dafang County, No. 7 Middle School, Bijie, Guizhou China; 4 Biostatistics and Research Methodology Unit, School of Medical Sciences, Universiti Sains Malaysia, Kubang Kerian, 16150 Kelantan Malaysia; 5 Hainan Normal University Sports Institute, Haikou, Hainan China

**Keywords:** COVID-19, Adolescents, Secondary students, NSMC, PL

## Abstract

Research indicates that COVID-19 has had adverse effects on the mental health of adolescents, exacerbating their negative psychological states. The purpose of this study is to investigate the impact of Physical Literacy (PL) on Negative Mental State caused by COVID-19 (NMSC) and identify potential factors related to NMSC and PL in Chinese adolescents. This cross-sectional study involved a total of 729 Chinese high school students with an average age of 16.2 ± 1.1 years. Participants’ demographic data, PL data, and NMSC data were collected. PL and NMSC were measured using the self-reported Portuguese Physical Literacy Assessment Questionnaire (PPLA-Q), the Stress and Anxiety to Viral Epidemics-6 (SAVE-6), and the Fear of COVID-19 Scale (FCV-19). Adolescents in the current study demonstrated higher levels of NMSC and lower PL, with average scores of 3.45 and 2.26, respectively (on a scale of 5). Through multiple linear regression analysis, Motivation (MO), Confidence (CO), Emotional Regulation (ER), and Physical Regulation (PR) were identified as factors influencing NMSC in adolescents. The study findings contribute to providing guidance for actions aimed at alleviating NMSC among adolescents.

## Introduction

Since the initial outbreak of COVID-19 in December 2019, the virus has shown a remarkably high transmission rate, causing significant global impacts. According to data from the World Health Organization (WHO) COVID-19 Dashboard in 2022 [1], the total number of confirmed global cases had exceeded 600 million by September. The ongoing mutations of the virus have led to sustained high numbers of confirmed and suspected cases, resulting in a prolonged coexistence with the virus. This situation has led to significant psychological distress and anxiety among individuals worldwide, disrupting normal lives [2, 3]. Despite a decline in the severity of COVID-19 over time and improvements in epidemic prevention and control worldwide, complete eradication of the virus remains elusive. The virus continues to pose a substantial threat to humanity, as noted by the WHO in 2023. The risk of new variants emerging and future waves of infections remains a genuine concern [4]. Recent data from the WHO’s COVID-19 Dashboard, as of June 7, reports a global death toll of 6,941,095 and a total of 767,750,853 confirmed cases. The trajectory of the outbreak has displayed unpredictable fluctuations in weekly confirmed case numbers worldwide since its inception, with a significant surge occurring in December 2022. Within just four weeks, this outbreak contributed to an increase of 97,976,070 cases globally (WHO, 2022). Numerous psychological effects have been studied in relation to the virus outbreak, ranging from personal to national and international levels [5]. On an individual level, people have experienced heightened fears of illness or death, a sense of helplessness, and the stigma associated with the virus, which collectively have led to severe mental health crises [6, 7]. The WHO has additionally reported that the global prevalence of anxiety and depression has increased by 25% due to the COVID-19 pandemic [8]. These findings underscore the persistent threat posed by COVID-19. To adequately address the potential for future recurrences of the pandemic, it is imperative to comprehensively understand the diverse psychological impacts of COVID-19 and develop effective response strategies.

Adolescents face unique challenges compared to working adults, often exhibiting emotional instability under pressure [9–11]. Their psychological characteristics set them apart from the general population and even from other young individuals, making them more vulnerable to psychological issues [12, 13]. The external environment has a stronger influence on them, increasing their susceptibility to problems like anxiety, depression, and post-traumatic stress disorder (PTSD) [14]. Their academic pressures and challenges are more pronounced, intensifying concerns about academic performance and future job prospects [15]. Unlike adults, their relative lack of experience in time management makes them more susceptible to the negative effects of remote learning [16]. A significant portion of students’ social and interpersonal interactions takes place on campus, and the isolation imposed by COVID-19 severs many of these connections. This isolation leads to increased loneliness and declining mental health, and these effects can persist for an extended period, not dissipating quickly even as the epidemic concludes [17]. Moreover, a substantial body of research indicates that quarantine measures during the pandemic have resulted in reduced outdoor activity, leading to decreased physical activity levels and a decline in physical control and motor skills [18, 19]. These issues can indirectly contribute to the emergence of psychological problems. Numerous studies have consistently demonstrated that the COVID-19 pandemic has had a profoundly detrimental impact on the mental well-being of adolescents [20–22]. This impact is characterized by a significant escalation in abnormal states of stress, anxiety, and fear, coupled with a noteworthy decline in the quality of sleep [23–25]. The multifaceted challenges posed by the pandemic, including disruptions to daily life, uncertainties about the future, and social isolation measures, have collectively contributed to the exacerbation of psychological distress among this demographic [26, 27]. It is imperative to recognize and address these mental health consequences to ensure comprehensive support for the well-being of young individuals navigating the complexities imposed by the ongoing global health crisis. Furthermore, the negative effects of COVID-19 can endure in terms of independence and social identity [28]. Addressing the psychological challenges faced by adolescents due to the COVID-19 epidemic has become an urgent research concern [29].

Several studies suggest that developing a stronger sense of self-efficacy can act as a protective factor against the adverse impact of challenging and stressful life situations, mitigating emotional distress and health-related risks [30]. Additionally, research indicates that self-efficacy serves as a negative predictor for symptoms such as depression and anxiety [31]. To assess the psychological dimension of participants’ physical literacy, the PPLA-Q (Psychological domain) scale was employed, encompassing four distinct factors: motivation, confidence, emotional regulation, and physical regulation. Notably, within the “confidence” aspect, it is including the task self-efficacy and barrier self-efficacy [32]. Thus, it is reasonable to hypothesize that strengthening PL has the potential to alleviate negative psychological states. Moreover, research suggests that enhancing the Physical Literacy (PL) of adolescents can contribute to increased motivation for engaging in physical activities, bolstered confidence levels, and improved abilities in emotional regulation [32]. Importantly, these enhanced abilities have a direct association with reduced levels of depression, anxiety, and stress, thereby promoting overall psychological well-being [33, 34]. Following this line of thought, we hypothesize that Physical Literacy may exert a negative predictive influence on the negative psychological repercussions induced by the COVID-19 pandemic.

## Method

### Participants

The study was granted ethical approval by the Human Research Ethics Committee of Universiti Sains Malaysia (USM) under the code USM/JEPeM/22,040,247. This ethical clearance is valid from August 21st, 2022, until August 20th, 2023.

This cross-sectional study was conducted from September to October 2022 in secondary schools located in Lanzhou city, Gansu Province, China. In Lanzhou city, there are a total of 80 high schools. To minimize the impact of residential areas and environments on the research results, this study divided the city into five regions: East, West, South, North, and Central. One school was then randomly selected from each region as a survey collection point. Within each of the five selected schools, two to three classes were randomly chosen through a lottery system to participate in the survey. This approach aims to ensure a representative and unbiased distribution across different geographical areas of Lanzhou, contributing to the robustness and generalizability of the research findings. All eligible students from the selected classes were invited to participate, resulting in a total of 729 participants. The average age of the participants was 16.2 years (SD = 1.1), with 493 (67.6%) being male and 236 (32.4%) being female. All participants were of Chinese nationality.

Inclusion criteria for this study were as follows: enrollment in a secondary school in Lanzhou city, age between 14 and 18 years, and proficiency in reading and communicating in Chinese. Students attending specialized schools were excluded from participation, as were those with visual, auditory, or other impairments that hindered their ability to independently complete the questionnaire.

### Instrument

#### Stress and anxiety to viral Epidemics-6 (SAVE-6)

The SAVE-6 questionnaire, developed by Chung et al. [35] in South Korea, is designed to gauge the stress and anxiety reactions of the general public in response to COVID-19. Consisting of six questions, it addresses key symptoms of stress and anxiety triggered by the pandemic. Following its use in Korea, the SAVE-6 scale demonstrated favorable internal consistency (Cronbach’s Alpha = 0.815). The scale has been recognized as a reliable, valid, and straightforward measurement tool suitable for assessing stress and anxiety in the general population.

#### Fear of COVID-19 scale (FCV-19)

The FCV-19 was created by Ahorsu et al. [36] to assess the fear, concern, and anxiety experienced by individuals towards COVID-19. This questionnaire includes seven items that probe public apprehension about the virus (item-total correlation ranging from 0.47 to 0.56). The scale exhibits solid psychometric properties, boasting acceptable reliability metrics such as internal consistency (α = 0.82) and test-retest reliability (ICC = 0.72). With its robust reliability and validity, the FCV-19 Scale proves valuable in evaluating COVID-19-related fears among the general public.

### Portuguese Physical Literacy Assessment Questionnaire (PPLA-Q)

The PPLA-Q, introduced by Mota et al. (2021), is tailored for assessing physical literacy among secondary school students. The questionnaire encompasses three cognitive, psychological, and social modules. In this study, the psychological module (consisting of 46 Likert-type items) is chosen. Participants evaluate their feelings on a five-point scale (ranging from “not at all” to “totally”), with scores assigned (0–4). The questionnaire has proven its effectiveness in measuring physical literacy among secondary school students in Portugal.

### Statistical analysis

All statistical analyses were conducted using IBM SPSS version 26.0 (IBM Corp., Armonk, NY, USA). Descriptive statistics were used to analyze the demographic data of high school students as well as the data for each factor of PL and NMSC. Simple linear regression was employed to examine the relationships between each predictor variable and the outcome variables (i.e., Motivation, Confidence, Emotional Regulation.

, Physical Regulation, and NSMC). The regression models utilised in the analysis aimed to provide a detailed understanding of the relationships between various predictor variables and the outcome variables, with particular emphasis on the justification for the inclusion of specific variables. A *p* value of 0.05 was set as the cut-off for statistical significance. Interactions, multicollinearity, model fitness and assumptions, outliers and influential cases were checked. Regression coefficients (b), 95% confidence intervals (CI), *p*-values, and overall coefficient of determination (r^2^) values were presented in the final model. These outcomes not only illuminated the magnitude and direction of the relationships between the predictor variables and the outcome variables but also provided a compelling rationale for the inclusion of specific variables within the regression analysis.

## Results

### Socio demographic characteristics of the study variables

As indicated in Table 1, the study comprised a total of 729 participants with an average age of 16.2 years (SD = 1.1), with a majority being male. All participants were of Chinese nationality. A substantial proportion of participants (60.5%) were below the age of 16, including 420 first-year high school students, 124 s-year high school students, and 185 third-year high school students.

**Table 1:** Demographic characteristics of people with participants (*n* = 729)

Characteristics		Frequencies	Percentage	Mean (SD)
**Gender**				
Male		493	67.6%	
Female		236	32.4%	
**Age**				16.2(1.1)
**Age group**				
Age 16 and under		441	60.5%	
Over 16 years old		288	39.5%	
**Grade**				
1		420	57.6%	
2		124	17.0%	
3		185	25.4%	

Table 2 displays the mean scores for each component scale and each factor of PL. Higher scores on the PPLA-Q indicate better PL in adolescents, while higher NMSC scores indicate poorer psychological states in adolescents. Motivation (MO), Confidence (CO), Emotional Regulation (ER), and Physical Regulation (PR) represent the four latent factors of PL. The mean (SD) scores for NMSC, PL, MO, CO, ER, PR were 3.455 (0.899), 2.265 (0.651), 2.223 (0.849), 2.242 (0.847), 2.322 (0.837), and 2.271 (0.822), respectively. The Composite Reliability scores for all latent factors were above 0.80.

**Table 2:** Mean scores of each questionnaire and factor

	PPLA-Q(PL)	SAVE-6	FCV-19	NMSC	MO	CO	ER	PR
Mean	2.265	3.439	3.469	3.455	2.223	2.242	2.322	2.271
Standard deviation	0.651	1.126	0.991	0.899	0.849	0.847	0.837	0.822
Score range	1–5	1–5	1–5	1–5	1–5	1–5	1–5	1–5
Minimum value	1.08	1.00	1.14	1.31	1.00	1.00	1.00	1.00
Maximum value	3.85	5.00	5.00	4.85	4.70	4.40	4.40	4.30

Note. MO = Motivation, CO = Confidence, ER = Emotional Regulation, PR = Physical Regulation

### Relationship between physical literacy and negative mental state caused by COVID-19

According to the hypothesis, PL can cause changes in NMSC, so PL is an independent variable. NMSC is the dependent variable. Physical Literacy includes MO, CO, ER and PR. The relationship between each factor and NMSC was verified by Regression analysis, and the results were shown in Table 3.

**Table 3:** Regression analysis of negative mental state and physical literacy

Variables	Simple linear regression			Multiple linear regression		
	Adjust b (95%CI)	t-value	*p*-value	Adjust b (95%CI)	t-value	*p*-value
Motivation	-0.419 (-0.514, -0.374)	-12.448	< 0.001	-0.153 (-0.241, -0.082)	-3.987	< 0.001
Confidence	-0.477 (-0.575, -0.439)	-14.651	< 0.001	-0.287 (-0.381, -0.228)	-7.822	< 0.001
Emotional Regulation	-0.424 (-0.526, -0.384)	-12.607	< 0.001	-0.174 (-0.267, -0.106)	-4.55	< 0.001
Physical Regulation	-0.398 (-0.509, -0.363)	-11.713	< 0.001	-0.105 (-0.198, -0.032)	-2.722	0.007

b = regression coefficient; *r*^2^ = 31.6%; stepwise, backward and forward methods applied; there were no interactions among independent variables; no multicollinearity detected; model assumptions are fulfilled

The provided table demonstrates the relationship between independent variables (MO, CO, ER, PR) and the dependent variable, NMSC, using linear regression analysis. The model’s R-squared value is 0.316, indicating that MO, CO, ER, PR collectively explain 31.6% of the variation in Negative Mental State caused by COVID-19.Furthermore, the model passed the *F* test (*F* = 83.813, *p* < 0.001, *df* = 4,724), suggesting that at least one of the independent variables (MO, CO, ER, PR) has a significant impact on NMSC.To address concerns of multicollinearity, all Variance Inflation Factor (VIF) values in the model were found to be less than 5, indicating the absence of collinearity issues. Additionally, the Durbin-Watson (D-W) value is close to 2, indicating no autocorrelation in the model and no correlation between the sample data, signifying a robust model.In a more detailed analysis: The standardized regression coefficient for MO is -0.153 (*t* = -3.987, *p* < 0.001), demonstrating a significant negative effect of MO on NMSC.The standardized regression coefficient for CO is -0.287 (*t* = -7.822, *p* < 0.001), revealing a significant negative effect of CO on NMSC.The standardized regression coefficient for ER is -0.174 (*t* = -4.550, *p* < 0.001), indicating a significant negative effect of ER on NMSC.The standardized regression coefficient for PR is -0.105 (*t* = -2.722, *p* < 0.001), suggesting a significant negative impact of PR on NMSC.In summary, MO, CO, ER, and PR were found to have significant negative effects on NMSC.

## Discussion

The PL has received relatively little attention in the realm of health promotion, particularly in the context of mental health. In fact, the study by Melby et al. [37] appears to be the sole exploration of the relationship between PL and adolescent mental health, underlining a substantial gap in our comprehension of the potential advantages of PL concerning mental health outcomes. This study endeavors to bridge this void in the literature by investigating the correlation between PL and NMSC in adolescents. Our hypothesis posited a negative correlation between PL and NMSC. The study’s significance lies in its potential to guide health promotion strategies, especially within educational settings where adolescents spend a substantial portion of their time.

This research delves into the sociodemographic characteristics of Chinese adolescents, specifically focusing on high school students from Lanzhou City, Gansu Province, China, with an average age of 16.2 years. The majority of participants, constituting 60.5% of the total sample, were under the age of 16.

Similar to other investigations [1, 28], the majority of our study’s participants exhibited high NMSC scores, as assessed by SAVE-6 and FCV-19. This implies that the COVID-19 pandemic has indeed amplified stress, anxiety, and fear among teenagers. Furthermore, the lower scores on the PPLA-Q signify weaker PL among the participants. PPLA-Q gauges PL across four latent factors: Motivation, Confidence, Emotional Regulation, and Physical Regulation. Consequently, our model incorporates these four independent variables, collectively influencing the Negative Mental State brought on by COVID-19, as evidenced by the significant F-test outcome (F = 83.813, *p* < 0.001). Subsequent examination of the regression coefficient values for each variable led us to conclude that MO, CO, ER, and PR all exerted significant negative effects on the NMSC. This suggests that all potential independent variables within PL contribute to NMSC, providing substantiation for the assertion that enhancing individual PL can effectively mitigate NMSC, thereby supporting our hypothesis.

Existing research suggests that promoting physical activity and PL among adolescents plays a pivotal role in enhancing their mental health [38]. Consequently, it can be argued that by promoting PL, individuals may be more inclined to engage in regular physical activity, potentially leading to improvements in their mental health outcomes and the alleviation of NMSC.

In summary, enhancing PL can augment an individual’s MO, CO, ER, and PR concerning physical activity. Despite the growing academic attention towards PL, its practical implementation and integration into policies have progressed at a comparatively slower pace. Nonetheless, its popularity is expected to surge in the forthcoming years [39]. Hence, advocating for PL and incorporating it into interventions aimed at bolstering mental health appears to be a promising avenue for enhancing the physical and mental well-being of adolescents.

It is crucial to acknowledge that the generalizability of the study’s findings is restricted. The study’s population consisted of young individuals from China, specifically from standard high schools in Lanzhou City. Further research encompassing diverse regions and youth populations, such as those in vocational high schools, is imperative. Furthermore, all survey data relied on self-reports from participants, which may introduce self-cognitive biases that could impact data precision. Additionally, participants may have been susceptible to social desirability bias, potentially leading to responses that align with perceived social expectations [40]. Efforts were made to encourage candid responses to mitigate this influence. In future research endeavors, researchers contemplate enhancing the credibility of the findings by incorporating physiological indicators associated with mental health. The inclusion of relevant physiological measures can provide additional evidence and strengthen the overall persuasiveness of the study outcomes. Introducing such physiological metrics may offer a more comprehensive understanding of the intricate interplay between physical literacy and mental well-being, contributing to a richer and nuanced interpretation of the research results. This expansion of methodology holds the potential to deepen insights into the mechanisms underlying the relationship between physical literacy and psychological health, fostering a more holistic approach to the investigation.

## Conclusion

In summary, this research illuminates the intricate relationship between PL and NMSC. The findings reveal that Chinese adolescents often exhibit lower levels of PL and heightened instances of NMSC. Notably, our study provides compelling evidence supporting an inverse correlation between PL and NMSC, underscoring the pivotal role of fostering PL to bolster mental health and overall well-being among this demographic. Furthermore, our research emphasizes the critical importance of promoting PL in adolescents, highlighting how engagement in physical activities to enhance PL can effectively alleviate NMSC. Essentially, our study contributes valuable insights into the potential benefits of advocating for PL to support the mental health and well-being of adolescents. Integrating PL into interventions aimed at enhancing physical activity and mental health outcomes emerges as a crucial strategy for cultivating a more active and mentally resilient younger generation. These findings not only deepen our understanding of the link between PL and mental health but also underscore the significance of targeted interventions to enhance PL in adolescents, paving the way for comprehensive approaches that contribute to both physical and mental well-being.

## Data Availability

Data will be made available upon request from WJ and GK.
